# Effect of Artemether-Lumefantrine Policy and Improved Vector Control on Malaria Burden in KwaZulu–Natal, South Africa

**DOI:** 10.1371/journal.pmed.0020330

**Published:** 2005-10-04

**Authors:** Karen I Barnes, David N Durrheim, Francesca Little, Amanda Jackson, Ushma Mehta, Elizabeth Allen, Sicelo S Dlamini, Joyce Tsoka, Barry Bredenkamp, D. Jotham Mthembu, Nicholas J White, Brian L Sharp

**Affiliations:** **1**Division of Clinical Pharmacology, University of Cape Town, Cape Town, South Africa,; **2**Health Protection, Hunter New England Population Health, Newcastle, New South Wales, Australia,; **3**Department of Statistical Sciences, University of Cape Town, Cape Town, South Africa,; **4**Malaria Research Lead Programme, Medical Research Council, Durban, South Africa,; **5**Malaria Control Programme, KwaZulu–Natal Provincial Department of Health, South Africa,; **6**Faculty of Tropical Medicine, Mahidol University, Bangkok, Thailand,; **7**Centre of Vaccinology and Tropical Medicine, Churchill Hospital, Oxford, United Kingdom; University of LondonUnited Kingdom

## Abstract

**Background:**

Between 1995 and 2000, KwaZulu–Natal province, South Africa, experienced a marked increase in *Plasmodium falciparum* malaria, fuelled by pyrethroid and sulfadoxine-pyrimethamine resistance. In response, vector control was strengthened and artemether-lumefantrine (AL) was deployed in the first Ministry of Health artemisinin-based combination treatment policy in Africa. In South Africa, effective vector and parasite control had historically ensured low-intensity malaria transmission. Malaria is diagnosed definitively and treatment is provided free of charge in reasonably accessible public-sector health-care facilities.

**Methods and Findings:**

We reviewed four years of malaria morbidity and mortality data at four sentinel health-care facilities within KwaZulu–Natal's malaria-endemic area. In the year following improved vector control and implementation of AL treatment, malaria-related admissions and deaths both declined by 89%, and outpatient visits decreased by 85% at the sentinel facilities. By 2003, malaria-related outpatient cases and admissions had fallen by 99%, and malaria-related deaths had decreased by 97%. There was a concomitant marked and sustained decline in notified malaria throughout the province. No serious adverse events were associated causally with AL treatment in an active sentinel pharmacovigilance survey. In a prospective study with 42 d follow up, AL cured 97/98 (99%) and prevented gametocyte developing in all patients. Consistent with the findings of focus group discussions, a household survey found self-reported adherence to the six-dose AL regimen was 96%.

**Conclusion:**

Together with concurrent strengthening of vector control measures, the antimalarial treatment policy change to AL in KwaZulu–Natal contributed to a marked and sustained decrease in malaria cases, admissions, and deaths, by greatly improving clinical and parasitological cure rates and reducing gametocyte carriage.

## Abstract 2

In KwaZulu-Natal strengthening of vector control and a change in antimalarial treatment policy to use of artemether-lumefantrine has been associated with a decrease in malaria cases, admissions, and deaths.

## Introduction

Malaria morbidity and mortality in Africa has risen, principally because of increasing resistance to chloroquine and sulfadoxine-pyrimethamine (SP) in *Plasmodium falciparum* [1,2]. Highly successful malaria control in South Africa, before the mid–1990s, had been achieved through high coverage with effective indoor residual spraying (IRS) and early access to effective antimalarial treatment. In KwaZulu–Natal in 1988, SP officially replaced chloroquine (CQ) as first-line treatment of uncomplicated malaria. In 1996, rapid immunochromatographic card tests were implemented to ensure definitive malaria diagnosis in all public-sector health-care facilities. Between 1995 and 2000 there was a dramatic increase in malaria morbidity and mortality in KwaZulu–Natal (Figure 1). Reinvasion of the area by the highly anthropophilic vector *Anopheles funestus,* which was resistant to pyrethroids, and a rapid increase in SP resistance in *P. falciparum* were considered the main contributors to this epidemic [3–5].

**Figure 1:**
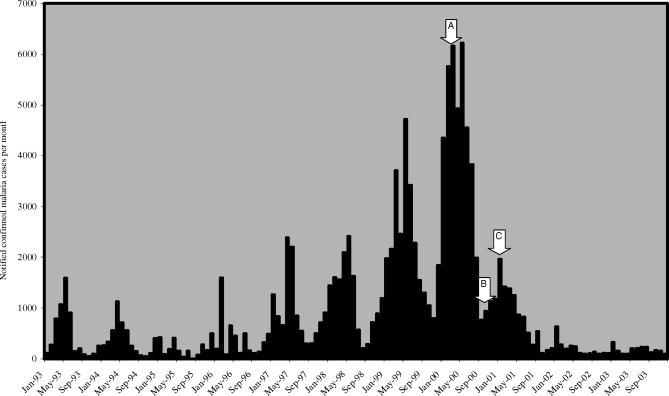
Number of Notified Malaria Cases in KwaZulu–Natal by Month (January 1993–December 2003) The number of cases is given in relation to season (peak transmission from January to May, inclusive) and timing of significant malaria control interventions: *A* indicates reintroduction of DDT for IRS of traditional structures in KwaZulu–Natal in March 2000; *B* indicates introduction of IRS in southern Mozambique in October 2000; and *C* indicates implementation of AL as first-line treatment of uncomplicated *falciparum* malaria in KwaZulu–Natal in January 2001.

To improve control of the *A. funestus* vector, an effective insecticide, dichlorodiphenyltrichloroethane (DDT), was reintroduced in March 2000 to replace failing pyrethroids for IRS of traditional (mud, reed, or wood) homesteads*.* However, western style structures, which constitute at least 40% of homesteads in the study area, continued to be sprayed with the pyrethroids because the residue left by DDT on painted or cement-plastered surfaces is aesthetically unacceptable. By 2003, *A. funestus s.s*. was identified in only three sites in northern KwaZulu–Natal [6], whereas *A. arabiensis* continues to be found sporadically in window exit traps throughout northern KwaZulu–Natal.

By 2000 the 42-d cure rate after antimalarial treatment with SP had fallen to 11% [5]. In response to this drug-resistant malaria epidemic, KwaZulu–Natal was the first Ministry of Health in Africa to implement an artemisinin-based combination therapy (ACT) as first-line treatment of uncomplicated *P. falciparum* malaria. The decision to adopt an ACT policy was influenced by the sustained high cure rates, decreased malaria transmission, and decreased antimalarial resistance that had been documented on the western border of Thailand [7]. It was supported by a growing international consensus that wide-scale systematic implementation of ACT is one of few effective measures that could enable malaria-endemic countries to achieve the ambitious goals set in Abuja to “Roll Back Malaria,” particularly the halving of malaria morbidity and mortality by 2010 [8]. Improved cure rates and decreased gametocyte carriage had been confirmed in recent large randomised controlled clinical trials conducted across Africa [9,10]. Decreased gametocyte carriage after AL treatment has been shown to reduce post-treatment transmission of *P. falciparum* to *Anopheles* mosquitoes [11]*.*

Artemether-lumefantrine (AL; Coartem, Novartis, Kempton Park, South Africa) was the only ACT available in 2000 with sufficient data to support fast-track registration by the South African drug regulatory authority. High levels of resistance in *P. falciparum* to CQ, SP, and amodiaquine in KwaZulu–Natal precluded their use in artemisinin-based combinations [5,12].

Although at least 14 African countries have recently adopted an ACT malaria treatment policy and an increasing number are in the process of changing to ACTs because of high levels of resistance to CQ and SP (the traditional first-line antimalarials) [13], concern has been expressed that the benefits of ACT observed in Asia have not yet been proven in Africa and may be influenced by coverage, adherence, quality, affordability, and access issues [14–17]. International subsidy of ACT costs has been widely recommended to address affordability constraints, although supply and quality issues currently remain substantial obstacles [18–20].

This study is a comprehensive evaluation of the first programme-wide implementation of ACT in Africa, describing changes in clinical and parasitological cure rates, gametocyte carriage, community perspectives on malaria treatment, and the impact of this ACT deployment and the strengthening of vector control on the number of malaria cases, admissions, and deaths. The factors considered to have contributed to the observed changes in morbidity and mortality are described.

## Methods

### Study Site

KwaZulu–Natal province, which has approximately 600,000 people living in malaria-risk areas, experienced the highest intensity malaria transmission in South Africa before 2001. Malaria risk and distribution is monitored through the provincial malaria geographic information systems that record all notified malaria cases, and provincial records are collated nationally [21]. Malaria transmission in South Africa has been restricted over the past four decades to the northeastern border areas with Mozambique, Swaziland, and Zimbabwe, primarily as a result of annual widespread IRS with insecticides by the provincial malaria control programmes, just prior to the malaria transmission season [22] and early effective treatment. Historically, the main mosquito vectors were the *A. funestus* group, which was considered to have been eradicated by IRS, and *A. arabiensis*, an efficient vector that displays both indoor and outdoor biting and resting behaviour, and thus is less readily controlled by IRS [23]. Following the identification of *A. funestus* resistant to pyrethroids in 1999 [3], traditional structures were sprayed with DDT (2 g/m^2^) although a pyrethroid (deltamethrin 0.02 g/m^2^) had to be applied to western-style structures.

Malaria transmission in KwaZulu–Natal is low (annual entomological inoculation rate <1; B. Sharp, unpublished data) and seasonal (Figure 1) [22]. The four sentinel health-care facilities studied, namely Ndumo clinic and Mosvold, Manguzi, and Bethesda rural district hospitals, are all located in Umkhanyakude district, which bears the heaviest malaria burden in KwaZulu–Natal (Figure 2). *P. falciparum* accounts for the majority (85%–100%) of infections.

**Figure 2:**
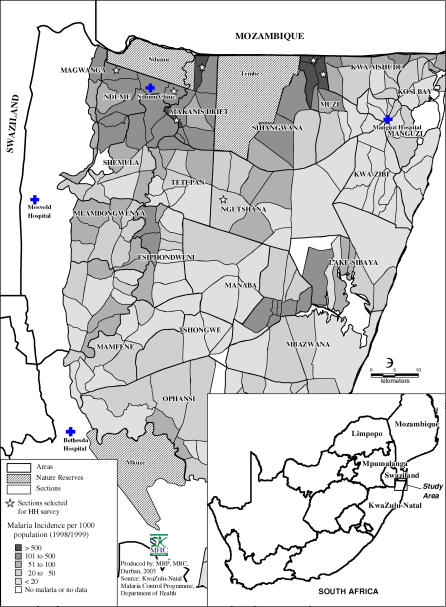
Map of Umkhanyakude District, Northern KwaZulu–Natal, South Africa The map indicates the following: the malaria risk by section and the four sentinel facilities for malaria morbidity and mortality review (Ndumo clinic, and Mosvold, Manguzi, and Bethesda rural district hospitals); the communities selected for the household (HH) survey and FGDs; and the Manguzi district hospital where sentinel safety surveillance and Ndumo Clinic where the SP (2000) and AL (2002) in vivo therapeutic efficacy studies were conducted.

South Africa has a decentralized health-care system. Antimalarial resistance patterns vary geographically. Malaria treatment policies have differed between provinces since 1988, when KwaZulu–Natal introduced SP to replace failing CQ, nine years before SP was introduced in the two other South African provinces with malaria transmission.

AL was implemented officially as first-line treatment of uncomplicated malaria in KwaZulu–Natal's public-health sector during January 2001 [24]. Malaria treatment in South Africa is administered on the basis of a *P. falciparum*–positive malaria smear or rapid immunodiagnostic card test. These tests are performed on all patients in whom malaria is clinically suspected; diagnostic quality control is regularly assessed by the Department of Health and the National Health Laboratory Services. Malaria treatment is provided free of charge in public-sector health-care facilities. AL was implemented 6 mo after the high SP failure rates were detected, and this delay consisted of a 3-mo policymaking process and a further 3-mo implementation preparation phase. AL was distributed to all public-sector fixed clinics (*n* = 50) and district hospitals (*n* = 5) in Umkhanyakude district in 24-tablet individual patient blister packs, for administration according to age–weight categories in a six-dose regimen over 3 d. Quinine remained the recommended treatment for severe malaria and for uncomplicated malaria in pregnant women and infants under 1 y of age. The implementation of AL included face-to-face training of public-sector health-care providers and distribution of specific malaria treatment guidelines and wall charts, and was followed by systematic withdrawal of SP.

### Facility Review of Malaria Cases, Admissions, and Deaths

Malaria morbidity and mortality data were collected retrospectively by reviewing hospital records provided by the medical superintendents between 2000 and 2003 at the sentinel clinic, Ndumo clinic, and the three sentinel hospitals, namely Manguzi, Mosvold, and Bethesda hospitals that serve Umkhanyakude district where the vast majority of malaria cases occur in KwaZulu–Natal (Figure 2). Malaria cases were defined as patients with clinical features of malaria in whom *Plasmodium* parasites were detected on malaria smear or rapid diagnostic test. Those malaria cases requiring admission for management of severe disease or that were considered a high-risk group, including infants and pregnant women, were classified as malaria-related hospital admissions. Malaria-related deaths were patients in whom malaria was included as a cause of death on the death certificate. Death-register data included those deaths that occurred outside the hospitals but were registered at a hospital for the purpose of issuing a death certificate required for burial.

### In Vivo Therapeutic Efficacy Study

Between January and June 2002, an open-label in vivo study was conducted to determine the therapeutic efficacy of a six-dose regimen of AL administered over 3 d (total adult dose 480 mg artemether/2,880 mg lumefantrine; Coartem, Novartis, Kempton Park, South Africa). Patients presenting sequentially to Ndumo clinic with an axillary temperature ≥ 37.5 ^°^C or a history of fever, who were older than 12 mo, weighed more than 10 kg, and lived close enough to the clinic to allow reliable follow-up, were screened for *P. falciparum* infection using a rapid immunochromatographic card test for detecting histidine-rich protein-2 (Malaria PF/PV ICTML02;SA Scientific Products, Midrand, South Africa). *P. falciparum* infection was confirmed by a positive Giemsa-stained thick blood smear. Informed consent was sought from all patients (or their guardians) with proven uncomplicated *falciparum* malaria and a parasite density between 1,000 and 500,000 asexual parasites/μl blood. Patients who reported receiving antimalarial treatment in the previous 7 d, pregnant women, or those with severe malaria [25] or danger signs (e.g. prostrate, repeated vomiting, dehydrated) were excluded. A pregnancy test (Human Chorionic Gonadotrophin Combo; Abbotts, Johannesburg, South Africa) was used to exclude pregnancy in any woman unsure of her pregnancy status.

Eligible subjects received the six-dose AL treatment according to body weight, and this was co-administered with 250 ml “amahewu” (a non-alcoholic fermented gruel-like drink made from maize and containing 0.3 g fat/100 g). The first, third, and fifth doses were administered under observation at the clinic. Patients were observed for 1 h following treatment and then allowed to return home. If vomiting occurred within 30 min of treatment, the patient was re-treated with a full dose. Vomiting between 30 and 60 min after treatment resulted in re-treatment with half the dose. Patients were advised to take the second, fourth, and sixth doses at home. Self-reported adherence with home treatment and timing of administration were recorded, together with pill counts, at each subsequent clinic visit. Concomitant medication without known antimalarial activity was administered at the investigator's discretion to relieve malaria symptoms or treat concurrent disease.

Clinical and parasitological response to treatment, and occurrence of adverse events were monitored on days 0, 1, 2, 3, 7, 14, 21, 28, and 42 according to modified World Health Organization (WHO) procedures [26]. Parasite clearance time was defined as the interval between initiation of treatment and the first of two consecutive negative thick blood smears. Filter-paper blood spots were collected for polymerase chain reaction (PCR) differentiation of re-infection from recrudescence using nested PCR amplifications of blocks within the polymorphic genes encoding glutamate-rich protein and merozoite surface proteins I and II [27]. Results were compared with those from a preceding open-label in vivo study of the therapeutic efficacy of a single dose of SP monotherapy (at a dose of 1.25 mg/kg pyrimethamine; Fansidar; Roche, Isando, South Africa), which followed a similar protocol and was conducted at the same site in 2000 with 98 patients completing 42-day follow-up [5].

Subjects were withdrawn from the study if they developed any danger signs of severe malaria [25], parasitological failure, a serious adverse event requiring withdrawal, or if the patient or their guardian requested withdrawal. “Rescue therapy” with quinine (quinine sulfate; Lennon Limited, Port Elizabeth, South Africa) was administered at a dose of 10 mg/kg three times daily for 7 d to patients with parasitological or clinical failure.

### Sentinel Pharmacovigilance

Intensive active sentinel surveillance for serious adverse events associated with antimalarial drug treatment was conducted at Manguzi hospital during and immediately after the malaria season (March–June 2002). This hospital was selected because it was the rural district hospital in the high-risk malaria transmission area with adequate resources to support intensive monitoring. The surveillance programme included any person presenting to Manguzi district hospital with a suspected serious adverse reaction after being treated with AL, or who was admitted for malaria, or had been treated for malaria in the 4 wk prior to hospital presentation. A standardised questionnaire was used to collect details of symptoms, clinical signs, and the results of special investigations for each patient. An international multidisciplinary panel, consisting of experienced clinical, laboratory, and public-health specialists (listed under acknowledgements), then reviewed the details of each case with any adverse effect to identify possible causal association with AL therapy. The total number of treatment courses distributed in the study area (Manguzi subdistrict) was used as a denominator for calculating the rates of adverse events.

In addition, all adverse events that occurred during the 42 d of follow-up in the in vivo therapeutic efficacy study were assessed to determine whether AL was a possible or probable cause according to guidelines of the International Committee for Harmonisation and the Medicines Control Council, the South African Drug Regulatory Authority [28,29]. These guidelines differ, in that lack of efficacy is classified as an adverse drug reaction in the Medicines Control Council guidelines, but not in the International Committee for Harmonisation guidelines.

### Household Surveys

Care-seeking behaviour for fever and malaria, and patient adherence with prescribed antimalarial treatment, were assessed using a survey questionnaire administered in the local language, isiZulu, to household members by specifically trained field teams between 6 to 12 wk after the implementation of AL. The highest risk communities within KwaZulu–Natal, with malaria notification rates ranging from 250 to 800 cases of malaria per 1,000 population in 1998–1999, were identified using the Malaria Control Programme Geographic Malaria Information System platform [21]. However, Muzi 2 section could not be accessed because of severe floods, and was replaced by Ngutshana 6, the section with the next highest malaria notification rates recorded in 2000–2001. All 439 households in the seven selected malaria sections were included in the survey (Figure 2).

Care-seeking behaviour was explored for three groups of respondents: those who reported being diagnosed with malaria in the previous 4 wk, those who reported an episode of fever (not considered by the respondent to be malaria) in the previous 4 wk, and those who reported “ever having had” malaria. Malaria diagnosis is generally definitively confirmed in patients seeking care at formal health-care facilities (rapid diagnostic testing is provided free of charge to both public- and private-sector facilities by the malaria control programme), but would be diagnosed clinically in those seeking treatment from traditional healers or not seeking treatment outside the home.

Patient adherence to antimalarial treatment was assessed by asking three main questions deliberately interspersed between other interview topics: (1) Do you have any treatment for malaria at home? (2) Do you still have any of this treatment (from this most recent malaria episode) remaining? and (3) Did you complete the malaria treatment course for your most recent infection?

### Focus Group Discussions

Four focus group discussions (FGDs) were conducted with 8–16 female household heads or caregivers in each of the areas selected for the household survey, to facilitate a greater understanding of community perspectives on treatment seeking and adherence to malaria treatment. The FGDs were conducted by an independent isiZulu–speaking qualitative researcher, in the home of a volunteering participant. Discussion topics included distinguishing between fever and malaria, management of children and adults with fever or suspected malaria, and factors influencing adherence with medication.

### Analysis

Data were double entered, verified, and, for the household survey, analysed using Microsoft Access 2000 (Microsoft, Redmond, Washington, United States). EpiInfo 3.01 (Centers for Disease Control, Atlanta, Georgia, United States) was used for analysing sentinel hospital and safety surveillance data. SPSS Version 8.0 (SPSS, Chicago, Illinois, United States) was used for analysing the in vivo study. Normally distributed continuous variables were compared using the *t-*test for independent samples, and proportions were compared using chi square tests (Yates' corrected or Fisher's exact, where appropriate), or odds ratios.

Stata 8.0 (College Station, Texas, United States) was used to analyse the gametocyte data. The cumulative transmissibility measure, the area under the gametocyte time curve (AUC), was calculated using the linear trapezoidal rule. Gametocyte density was compared between groups using the ratio of arithmetic means. This was done by fitting generalized linear mixture models with a logarithmic link function to model the logarithm of the (arithmetic) mean gametocyte density and a logistic link function to model gametocyte prevalence using a zero-inflated negative binomial distribution. This accounted for the typically skewed distributions and excess variance, and allowed inclusion of all data points (including zeroes), modified from the method of Sutherland et al. [11]. This model generated parallel results that provided estimates of the relative risk of a zero gametocyte density and estimates of the incidence rate ratio of the mean gametocyte densities in the two treatment groups [30].

FGDs were independently analysed by two authors (KIB and SSD) who identified consistent themes or analytical categories in the data using an iterative approach, based on an adaptation of the grounded theory approach [31]. FGD findings were confirmed by a third independent reviewer. The findings of the household survey and FGDs were compared for consistency, thus reducing the possibility of systematic distortions inherent in using only one method [32].

### Ethical Considerations

This study was approved by the Research Ethics Committees of the University of Cape Town and the South African Medical Research Council, and was planned and conducted in full partnership with the KwaZulu–Natal Ministry of Health. In the in vivo studies, written informed consent was obtained in the patients' local language, isiZulu, from all literate patients or guardians, and an independent literate witness confirmed verbal consent for illiterate patients or guardians who also recorded their consent as an “X” on the consent form. Verbal informed consent was obtained from participants in the household survey and FGDs. Confidentiality of patient identity was maintained for all records.

## Results

### Review of Malaria Cases and Deaths

The catchment areas of the three sentinel hospitals studied include almost 285,000 (47%) of the estimated 600,000 persons at risk of malaria in KwaZulu–Natal in 2000; these facilities carry the heaviest malaria burden because they are in the highest risk area in the far northeast of the province. There was a very marked reduction in the number of malaria cases, hospital admissions, and deaths in all these sentinel health-care facilities between 2000 and 2001, that were sustained (Table 1). Between 2000 and 2001 the number of malaria deaths and admissions both decreased by 89%, and malaria outpatient cases decreased by 85%. These initial reductions in malaria cases (96% versus 86%), admissions (92% versus 82%). and deaths (93% versus 78%) were greatest at Mosvold hospital and lowest at Manguzi hospital. Because Manguzi hospital borders on southern Mozambique, the migrant proportion of their patients may not have benefited to the same extent from the change in the first-line treatment policy in KwaZulu–Natal. The KwaZulu–Natal Department of Health estimates that Manguzi hospital serves a catchment population of approximately 20,000 people from southern Mozambique.

**Table 1:**
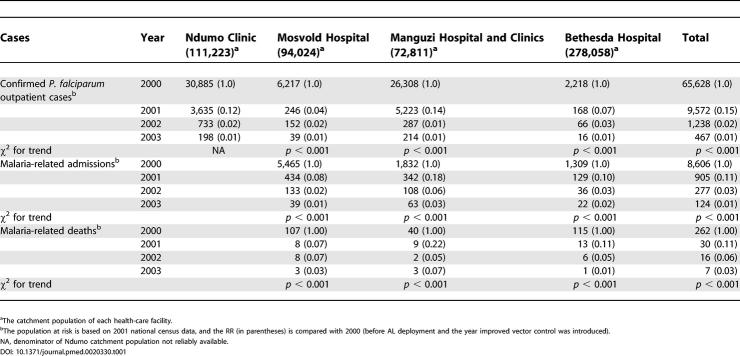
Confirmed Malaria Cases and Malaria-Related Hospital Admissions and Deaths in the Sentinel Health-Care Facilities with the Highest Incidence of Malaria in Northern KwaZulu–Natal, South Africa, between 2000 and 2003

The marked reduction in malaria morbidity and mortality continued at all sentinel facilities. By 2003 the number of malaria-related outpatient cases and admissions had decreased by 99%, and malaria-related deaths had decreased by 97%. Trend analysis showed a highly significant decrease at all sentinel hospitals (*p* < 0.001).

### In Vivo Study of Therapeutic Efficacy

Between January and June 2002, 100 patients (53 female) with uncomplicated malaria who sequentially met inclusion criteria were enrolled in this study. Patients had a median age of 14 y (interquartile range [IQR] 9–28 y) with 5% under 5 y of age. The geometric mean parasite density at baseline was 26,705/μl (95% confidence interval [CI]: 20,385 to 34,985). The median duration of symptoms at presentation was 3 d (range 0–14 d). Paracetamol (approximately 15 mg/kg) was administered at least once to 87% of patients. Antibiotics (without antimalarial activity) were administered to three patients in addition to AL. Two patients were lost to follow-up on day 28, and one patient missed his follow-up visit on day 28 but attended the day 42 visit. Parasites reappeared in one patient on day 28. Because the PCR results for this patient were indeterminate, this was assumed conservatively to be a recrudescence. There were no other parasitological failures and no clinical treatment failures. Of the 100 patients, two carried gametocytes after treatment; both these patients had gametocytes present in their blood smears at enrolment. No gametocytes developed in any patient in whom gametocytes were absent on day 0.

These results were compared with the in vivo study of the therapeutic efficacy of SP conducted in 2000 at the same sentinel study clinic, in a study population with a median age of 13 y (IQR 7–18 y) with 17% under 5 y of age.[6] The baseline geometric mean parasite density was significantly higher in the SP monotherapy study (62,114/μl [95% CI: 47,570–81,104]); *p* < 0.05. Pretreatment gametocyte carriage was 1/98 in 2000 and 3/100 in 2002 (*p* = 0.621). Parasite clearance time was significantly more rapid after AL (54 h [CI: 47–60]) than after SP (125 h [CI: 70–180]). Parasitological cure rates at 42 d of follow-up (corrected for PCR-confirmed reinfections) was 11/98 (11%) with SP monotherapy compared with 97/98 (99%) with AL in the present study (*p* < 0.001). Survival analysis confirmed markedly superior clinical and parasitological responses following AL, when compared with SP (log-rank test *p* < 0.001) (Figure 3). Early treatment failures decreased from 15/98 (15%) with SP to 0/98 with AL (*p* < 0.001) and late parasitological failures decreased from 72/98 (73%) to 1/98 (1%) (*p* < 0.001). Reinfections during follow-up decreased from 4/98 in 2000 to 0/98 in 2002 (*p* = 0.121).

**Figure 3:**
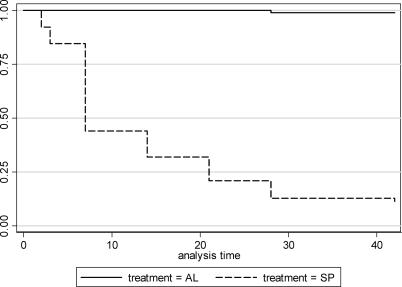
Kaplan-Meier Survival Analysis of Time to Clinical or Parasitological Failure Following treatment with SP in 2000 (*n* = 98) and artemether-lumefantrine in 2001 (*n* = 100), the proportion of patients with an adequate clinical and parasitological response to treatment at each day of follow up is shown.

During the 2000 SP in vivo therapeutic efficacy study, 52 (57%) of the 91 study subjects for whom gametocyte densities were recorded were found to carry gametocytes after treatment with SP compared to 2/100 (2%) after AL treatment (relative risk [RR]: 28.5 [95% CI: 7.16 to 113.97]); *p* < 0.001] (Figure 4). Gametocyte carriage peaked on day 7 following SP treatment, and on day 0 (prior to starting treatment) for AL. The median gametocyte area under the time curve (AUC) decreased significantly from 420 (IQR: 0–2,797) gametocytes/μl per person-week after SP to 0 (IQR: 0–0) gametocytes/μl per person-week after AL (Kruskal-Wallis *p* < 0.001). Regression analysis of the cumulative area under the gametocyte time curve found that the RR of a zero gametocyte density following treatment was 44-fold higher (95% CI: 13–148) after AL than SP, representing a 97.7% relative reduction in gametocyte prevalence (*p* < 0.001). In addition, AL was associated with a 95% decrease in gametocyte density among those carrying gametocytes (incidence rate ratio = 0.050; 95% CI: 0.009–0.274; *p* = 0.001).

**Figure 4:**
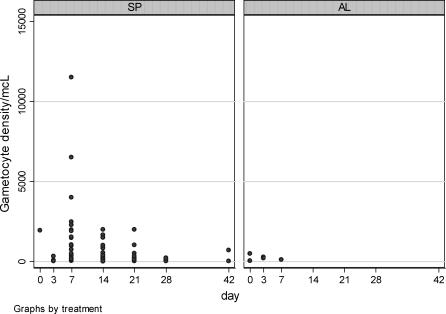
Scatter Plot of Individual Patient Gametocyte Densities The gametocyte densities (per microlitre) are given over time following treatment with SP (2000) and AL (2002).

### Safety Monitoring and Adverse Events

Of 17 adverse events reported by patients recruited into the in vivo study, four were considered probably related to AL, but none of these was considered serious. A case of urticaria on day 1 resolved with administration of an antihistamine (without discontinuation of AL). There were two cases of vomiting and one possible treatment failure on day 28. Four patients demonstrated a sustained decrease in haemoglobin (Hb), although this was considered of potential clinical significance in only one case (Hb = 93 g/l on day 0, Hb = 70 g/l on day 7, and Hb = 74 g/l on day 42). The Hb level remained above 100 g/l, despite a decrease of at least 20 g/l, in the other three patients. Mouth ulcers that affected three patients after AL, and pre-existing vomiting that persisted in two cases after AL, were considered possibly related to treatment. The remaining adverse events were considered more likely due to malaria (body pains, *n* = 2) or inter-current illness (chest pain which resolved with amoxicillin, *n* = 1; ear infection, *n* = 1).

From April to June 2002, when approximately 310 blister packs of 24 tablets were dispensed in this hospital's catchment area, a total of 44 patients admitted to Manguzi hospital met the inclusion criteria of the intensive hospital-based safety monitoring study. Two of these patients did not provide consent and were thus excluded from the study. The remaining 42 patients, of whom 12 (29%) were under 2 years of age, were admitted either for malaria (*n* = 36), or for persistent (*n* = 5) or new (*n* = 1) symptoms following antimalarial treatment.

A total of 13 patients were treated with AL before (*n* = 5) or during (*n* = 8) their hospital stay. The five patients who had recently been treated with AL were admitted because of a poor response to AL, although it was not confirmed whether or not these patients had adhered fully to this treatment. These would not be considered adverse drug reactions according to International Committee for Harmonisation criteria. One 14-y-old female patient died. She had apparently received AL 3–4 d prior to her death, and was then treated with an unidentified herbal mixture by a traditional healer when she did not improve. She was admitted with a diagnosis of possible cerebral malaria with herbal intoxication and died within 24 h of admission. On admission her malaria smear was negative and a cerebrospinal fluid examination confirmed bacterial meningitis; the multidisciplinary expert review team concluded that her death was unlikely to be related to AL. One 24-y-old female patient treated during hospital stay with AL and doxycycline experienced vomiting after receiving the first and subsequent dose; this was considered a possible adverse reaction to AL and/or doxycycline.

### Community Perspectives on Malaria Treatment Seeking and Adherence

Two of the 439 households selected for the household survey elected not to participate, and one respondent was unable to complete the interview due to illness (data from the portion of her interview completed was included in this analysis). Data were collected on a total of 2,506 household members of whom 55% were female. The average estimated distance from study households to the nearest public healthcare facility was 6.5 km, and this took an average of 90 min to reach. Overall 68% of household members had previously suffered from malaria.

Respondents reported that 239 (10%) household members had suffered from malaria in the 4 wk immediately preceding the household survey, which was conducted during February and March 2001 at the peak of the malaria transmission season, and 4 to 6 wk after the implementation of AL. Of these, only four (1.7%) reported not seeking treatment outside the home. Most had first sought treatment at either a Malaria Control Programme field camp (*n* = 101; 42%) or a public sector clinic (*n* = 127; 53%). Five patients reported initially seeking treatment at a public hospital, whereas one patient sought treatment from a private doctor, and one indicated first going to a traditional healer. Respondents reported that 226 (96%) had completed recent treatment (i.e., taken all prescribed tablets) but were uncertain whether a further seven (3%) household members had completed treatment. These responses were consistent with those regarding whether there was any antimalarial treatment remaining at home, with only two patients (1%) admitting that they still had antimalarial tablets (both AL) remaining from the recent treatment course.

Amongst household members that reported never having had malaria (“malaleveva”), 26% (204/785) reported experiencing fever (“umkhuhlane”) in the 4 wk prior to the household survey. Only 88 (43%) had sought care outside the home. Of those seeking fever treatment, 79 (90%) had presented to a public sector clinic, five at a public sector hospital, one to a private doctor, two to a Malaria Control Programme field camp, and one to a traditional healer. A significantly greater proportion of patients with recent perceived malaria (235/239; 98%) than those with a recent febrile disease not considered to be malaria (88/204; 43%) sought treatment at health-care facilities (RR: 2.28; CI: 1.95–2.67; *p* < 0.001).

Participants in all four FGDs agreed that if home treatment was not successful in rapidly alleviating malaria symptoms, the affected household member would be taken to the nearest public health-care facility. The FGDs revealed that people generally first attempted treatment of “just” fever with herbal medicines and enemas. Participants mentioned weakness and headache more often than fever as a feature they associate with malaria; participants commented that malaria, unlike “just” fever, was not self limiting. When asked about sources of treatment for malaria fever, all FGDs emphasized that there was no effective alternative to public health-care facilities and that malaria treatment should be sought urgently: “There's nothing else, you rush to the clinic” and “I have never heard of someone who cleared malaria at home, without going to the clinic.” Delaying treatment seeking was considered potentially fatal: “Once you don't go to the clinic, it kills…. You will die from malaria” and “Once you waste time at the sangoma [traditional healer], you die.”

The change in treatment policy from SP to AL appeared well accepted, because SP was regarded as ineffective: “These tablets (SP) which were used before the present ones [AL]—most of us didn't like taking them.…You would feel as if the malaria has become more severe,” “Every year I used to have malaria. This year I heard that new pills were coming. I was very sick with malaria. They gave me the [new] pills…. The [next] morning I was very fine…. I have cultivated and harvested and I've never had malaria [again] this year.” Although FGD participants generally reported completing full malaria treatment courses (“We finish the malaria treatment because we have seen that it [malaria] finishes [kills] us”), a few individuals indicated terminating treatment once symptoms resolved and saving remaining medication for future use.

## Discussion

Reversing the alarming increase in malaria associated mortality in Africa is possible with use of existing effective methods of vector control and widespread use of highly effective ACT. KwaZulu–Natal Province was experiencing an epidemic of malaria fuelled by re-emergence of an insecticide-resistant mosquito vector and spiralling resistance to SP. There was a dramatic response to strengthening the vector control programme and wide-scale implementation of an ACT, AL, in this low-intensity malaria transmission setting; the number of malaria cases fell by 99%. The implementation of these dual interventions in KwaZulu–Natal was found to be cost effective and resulted in substantial cost savings [33].

Malaria transmission is multifactorial: In KwaZulu–Natal, where malaria control operations are intense, exploration of monthly malaria case data between 1971 and 2001 found that climatic factors only influenced interannual variation in malaria transmission but not the medium to long term trends in case totals, which were significantly associated only with antimalarial resistance and HIV prevalence [34,35]. Despite the continued increase in antenatal HIV seroprevalence in KwaZulu–Natal from 32.5% in 1999 to 40.7% in 2004 (http://www.doh.gov.za/aids/index, we observed a 99% reduction in confirmed malaria cases. However, HIV coinfection may have precluded a greater reduction in malaria-related admissions and deaths because this coinfection has been shown to increase the risk of severe malaria and malaria-related deaths in the non-immune KwaZulu–Natal population [36]. There were no other substantial social, political, and health-care changes likely to affect malaria burden during the study period, although small effects cannot be excluded.

Notification of communicable diseases is constrained by under-reporting and this is particularly severe during epidemics in resource poor areas, when health workers probably prioritise patient management over notification [37]. The increased demand placed on health-care providers by the large-scale malaria epidemic in KwaZulu–Natal Province that peaked in 2000 suggests that under-notification may have been particularly prevalent during that year. It is expected that any delay in changing the SP malaria treatment policy and in strengthening vector control would have resulted in increased malaria transmission and further increases in malaria morbidity and mortality. Thus the remarkable reduction in malaria case notification documented here is probably an underestimate of the true effect of implementing effective treatment and improving vector control.

The decreases in malaria morbidity and mortality observed in KwaZulu–Natal Province reflect both the therapeutic effects of AL and the enhanced malaria vector control (following the reintroduction of DDT and extension of IRS to neighbouring southern Mozambique). The reliable and rapid therapeutic response to ACTs, combined with their effect of reducing gametocyte carriage, make them ideal treatments. The dramatic reductions in gametocyte prevalence and density observed would substantially contribute to reducing malaria transmission [11]. The relative contributions of the two strategies, deliberately introduced in short succession to optimise public health impact, cannot be accurately apportioned. The extent of the public health impact observed following the policy change to ACTs is unlikely to have been achieved in the absence of effective vector control. Equally, we believe that the benefits of reintroducing DDT for IRS of traditional (but not western-style) structures would have had less benefit without the replacement of highly ineffective SP with an effective acceptable ACT, given the 89% treatment failures and high gametocyte carriage rates observed following SP monotherapy.

On the northwestern border area of Thailand, a 47% reduction in the incidence of *P. falciparum* infections was observed in the 12 mo following the introduction of artesunate plus mefloquine, when no changes were made to vector control [38]. This improved further to a 6-fold reduction over a 10-y period of use [7]. The similar experience of marked public health benefits in northwestern Thailand suggests that the results in KwaZulu–Natal Province reflect the benefits of effective ACT, rather than being specific to AL. The area studied on the western border of Thailand is similar to KwaZulu–Natal in terms of a low intensity of seasonal malaria transmission (annual entomologic inoculation rate < 1) and reasonably high levels of access to health-care facilities providing definitive diagnosis of malaria and relatively reliable, well-regulated drug supply. The reduction in malaria mortality by ACTs reflects both the reduced incidence of malaria following decreased gametocyte carriage, and the reliable and rapid antimalarial activity of the ACT. General deployment of artemisinin in Vietnam was also associated with a marked and sustained reduction of malaria mortality [39].

The improvement in clinical and parasitological cure rate from 11% to 99% is particularly important in KwaZulu–Natal, because the low intensity of malaria transmission and, consequently, the low levels of acquired immunity mean that a substantial proportion of patients with parasitological failure would develop recrudescent *P. falciparum* infections or even progress to severe malaria. The high AL cure rate, similar to the 97%–98% published recently in large randomised controlled trials reported from East Africa [40,41], was achieved despite co-adminstration at the clinic with a relatively low-fat content drink, which had been selected for its widespread availability. Previous studies have shown that lumefantrine absorption is highly dependent on coadministration with fat, although the minimum fat content required for adequate absorption has not yet been defined [42].

Similarly to previous studies [43,44], we documented that AL was well tolerated both in the sentinel surveillance programme at Manguzi hospital, and in patients enrolled in the in vivo study who were closely monitored. This probably contributed to the generally good adherence reported. However, the markedly decreased malaria incidence in KwaZulu–Natal Province limited our ability to detect uncommon serious adverse effects of AL.

In South Africa, orthodox medicines are relatively strictly regulated, and AL treatment requires prescription by a registered health-care provider. ACT coverage depends on treatment seeking at health-care facilities where AL is available. Household survey results demonstrated that the majority (97%) of people with recent symptoms thought to result from malaria initially sought treatment at public health-care facilities. For recent fevers not considered by respondents to be related to malaria, however, seeking treatment outside the home was significantly less frequent, although the public clinics remained the most popular source of health care. These malaria-specific treatment-seeking patterns are likely to have been enforced by the regular communication between the provincial Malaria Control Programme and the community. The general preference for public health-care facilities may be associated with the provision of free health care for notifiable diseases in public sector clinics and a relatively high level of access to these facilities, with 81% of the population living within 10 km of a public clinic in northern KwaZulu–Natal [21]. This pattern of treatment-seeking behaviour was confirmed during the FGDs, in which fear of malaria and perceived effectiveness of treatment served as key factors motivating patients to seek treatment from public health-care facilities. High levels of AL coverage are thus achievable in northern KwaZulu–Natal through public-sector implementation alone, provided that patients recognise malaria symptoms. Because ACT coverage is considered an important determinant of community benefit in terms of reducing malaria transmission and slowing antimalarial resistance, ensuring ready access to this treatment (and definitive diagnosis) is a key component of effective malaria control. This cannot be achieved without addressing local health-care infrastructure needs and ensuring a high level of health literacy in the local community.

Adherence with prescribed antimalarial treatment regimens is essential to optimise cure rates and prevent resistance. There were some inconsistencies between household survey and FGD findings on patient adherence, with a few FGD participants indicating that they would stop treatment once symptoms resolved and save medication for future episodes. These FGD findings confirm, but cannot quantify, the general trend that self-reporting tends to overestimate levels of adherence, because patients who report poor adherence are generally accurate, whereas those who deny poor adherence may not be [45]. Although the consistently high levels of adherence reported in the answers to three different questions, and the dramatic reductions in malaria morbidity and mortality are reassuring findings, it has been suggested that the public health consequences of partial adherence are delayed until cure rates start to decline [46]. There is a clear need for better measures of adherence in general and, for AL in particular, for ongoing efforts by health staff to encourage patients to complete all six doses and to co-administer it with a fat-containing drink.

There are a number of factors to be considered before assuming direct generalisability of our findings to other countries, including: whether the current malaria treatment policy is already highly effective, whether baseline resistance to the non-artemisinin component of the ACT is higher, whether home treatment and treatment seeking in the informal or private sector is more prevalent, whether malaria diagnosis is clinical or confirmed, and perhaps most importantly, the intensity of malaria transmission intensity. Recent data suggest fewer than half of the populations at risk of malaria globally live in areas of high-intensity malaria transmission, with 25.4% of those at risk living in low-intensity transmission areas and a further 31.3% living in areas of moderate-intensity malaria transmission [47].

Decreasing malaria morbidity and mortality substantially in high-transmission areas, as in much of sub-Saharan Africa, is expected to be considerably more challenging. The higher risk of new malaria infections could impact on the overall effectiveness of ACTs, and ACT coverage and compliance may be lower in semi-immune individuals because a substantial proportion of infections would be asymptomatic and symptoms would more likely resolve with incomplete treatment. There are fewer examples of successful large-scale vector control programmes in high-intensity transmission areas.

Between the 1940s and 1960s, pilot malaria eradication projects across sub-Saharan Africa recorded significant reductions in malaria [48]. Subsequently, systematic indoor residual insecticide spraying programmes have been highly successful in reducing malaria transmission, particularly in southern Africa and island states [49]. Similarly, widespread coverage with insecticide-treated bed nets in areas of high-intensity malaria transmission has resulted in sustained protection of both individuals and communities against malaria [50]. These findings suggest that effective sustainable vector control may be achievable across all levels of malaria endemicity and could limit the number of malaria cases requiring ACT treatment, particularly if malaria is definitively diagnosed. This would increase the affordability of ACTs and adequacy of ACT supply, and be expected to optimise the effect of ACTs on malaria transmission.

### Conclusions

We consider the ready access to treatment in a relatively well-developed rural primary health-care infrastructure, coupled with an effective vector control programme, important factors for deriving the greatest benefit from ACT implementation. Equally important are the strong community perceptions that malaria diagnosis and treatment should be sought urgently at public health-care facilities and treatment then completed. These factors deserve consideration by those responsible for reducing malaria morbidity and mortality, because wide-scale implementation and rational use of effective vector control and ACT are cornerstones of combating the enormous health and economic burden of malaria.

**Patient Summary**

**Background**

Malaria is caused by a parasite transmitted by some types of mosquito; it kills about a million people every year, especially children in Africa. The disease has become more common in recent years because the parasites have become resistant to many malaria drugs and the mosquitoes have developed resistance to insecticides. Between 1995 and 2000, malaria increased dramatically in South Africa's KwaZulu–Natal province. The main reasons for the increase are believed to be resistance to the drug sulfadoxine-pyrimethamine (SP) and to pyrethroid types of insecticide. In 2000, in response, a new drug called artemether-lumefantrine (AL) was introduced to treat people with malaria. (AL is a combination drug that includes artemisinin, which has been shown to work in control programs in Asia, but this the first time a drug of this type has been used in a program in Africa.) Mosquito control efforts were also increased and, although pyrethroids were still used in some situations, the older insecticide DDT was also reintroduced.

**What Did the Researchers Do and Find?**

They measured the success of the action taken in KwaZulu–Natal. In the year following the changes, hospital admissions for malaria declined by 89% and so did deaths; outpatient cases decreased by 85%. By 2003, outpatient cases and admissions had both fallen by 99% and deaths had decreased by 97%. AL treatment cured 99% of cases, compared with the 11% cure rate previously found with SP. Most patients (96%) said they completed the six-dose course of AL, and no serious drug side effects were reported.

**What Does This Mean?**

The study shows an encouraging and important example of how malaria can be effectively fought. Because both better mosquito control and better drugs were introduced around the same time, the researchers could not say how much each of the two measures contributed to the overall success. However, it is likely that both contributed, and it shows that the two together can be very effective. When applying the lessons from this success story to other parts of Africa and to other continents, one needs to keep in mind that the starting conditions in KwaZulu–Natal were quite favorable. For example, there is a low level of background immunity against malaria, which means that infected people usually get quite sick and seek treatment rather than acting as reservoirs for further transmission. In addition, most patients can get prompt diagnosis and treatment because the province has a reasonable health-care infrastructure.

**Where Can I Get More Information?**

The Roll Back Malaria Department of the World Health Organization has a Web site with information about the disease and the global efforts to fight it (this site also has links to other organizations and to useful publications):

http://www.who.int/malaria

The South African Department of Health Web site includes guidelines on the treatment of malaria (2002) and prevention of malaria (2003):

http://www.doh.gov.za/docs/index.html

## Abbreviations

ACT: artemisinin-based combination therapy
AL: artemether-lumefantrine
CI: confidence interval
CQ: chloroquine
DDT: dichlorodiphenyltrichloroethane
FGD: focus group discussion
Hb: haemoglobin
IQR: interquartile range
IRS: indoor residual spraying
RR: relative risk
SP: sulfadoxine-pyrimethamine
WHO: World Health Organization
